# Nutritional deficiency post esophageal and gastric cancer surgery: A quality improvement study

**DOI:** 10.1016/j.amsu.2020.05.032

**Published:** 2020-05-29

**Authors:** Pushpa Veeralakshmanan, Ji Chung Tham, Amy Wright, Marilyn Bolter, Himanshu Wadhawan, Lee M. Humphreys, Grant Sanders, Tim Wheatley, Richard J. Berrisford, Arun Ariyarathenam

**Affiliations:** Peninsula Oesophago-gastric Centre, University Hospitals Plymouth NHS Trust, Derriford Road, Plymouth, PL6 8DH, United Kingdom

**Keywords:** Esophago-gastric cancer, Esophagectomy, Gastrectomy, Micronutrient deficiency, Allied health professionals

## Abstract

**Background:**

Esophagectomy or gastrectomy for malignant tumors can have a profound effect on nutritional status of patients undergoing the procedure. Hence, postoperative nutritional status is an important prognostic factor to consider in ensuring optimal recovery. In this study, we looked at assessing the prevalence of micronutrient deficiencies post esophagectomy or gastrectomies and the efficiency of Allied Health Professionals (AHP) led clinics in identifying and appropriately managing the deficiencies.

**Method:**

Between February 2017 and February 2018, all patients who attended the AHP clinic, had micronutrient screening, which includes ferritin, folate, vitamin B12 and vitamin D. Patients were screened for exocrine pancreatic insufficiency (EPI) through series of questions related to symptoms of EPI including steatorrhea, flatulence and urgency to defecate. All patients included in the study were started on A-Z multivitamin tablets from their first visit. Patients reporting symptoms indicative of EPI were started on Creon. Patients found deficient in any micronutrients were invited for a follow-up measurement of the respective deficiency.

**Results:**

A total of 63 patients were included in the study period with a median follow-up of 18 months (range: 2–60 months) post esophagectomy and/or subtotal/total gastrectomy for malignant tumors. Proportion of patients with deficiency in ferritin, folate, vitamin B12 and vitamin D were 42.86%, 9.52%, 6.35% and 36.67% respectively. The proportion of patients identified with symptoms indicative of EPI was 31.75%. At re-test follow-up, 66.67% patient noticed settlement of symptoms of EPI. Ferritin, Folate, Vitamin B12 and D levels significantly improved post initial AHP follow-up (significance level p < 0.05).

**Conclusion:**

This study highlights that nutritional deficiencies post esophagectomy and/or subtotal/total gastrectomy for malignant tumors are prevalent. AHP run follow-up clinics in our unit helps identify these deficiencies and manage them accordingly. This study shows statistically significant improvement in deficiencies thereby making AHP led follow-up clinics to be cost effective and improve patient outcome.

## Highlights

• In our study, 39.13% of the patients had >15% weight loss at 3 years post esophageal and/or gastric cancer surgery.
• Allied Health Professionals led clinics can provide safe and cost-effective screening for micronutrient deficiencies.
• Appropriately correcting the micronutrient deficiencies can enable patients to have a better quality of life.

## 1 Introduction

Esophageal cancer is the 13th most common cancer in the UK and accounts for up to 3% of all new cancers [1]. Due to advancements across various spectrum of care in esophagectomy patients, there has been a growth of focus towards nutritional status of those patients [2,3]. Surgery for upper gastrointestinal cancers can have a profound effect on nutrition. Several studies have reported deterioration in nutritional status post esophagectomy mostly after the first 6 months of surgery [2]. Most of these studies used change in weight (as a percentage or absolute amount) or body mass index (BMI) as their postoperative nutritional parameters [2]. However, actual measurement of serum nutrition levels could be a better method to assess nutritional status. Other recognized symptoms post esophagectomy or subtotal/total gastrectomy includes anorexia, diarrhea and dumping syndrome [3]. The etiologies for these symptoms are multifactorial and not fully understood. Malabsorption can be caused by exocrine pancreatic insufficiency (EPI) and small intestinal bacterial overgrowth (SIBO). SIBO occurs in these patients due to diminished gastric acid secretion, anatomical alterations of the gut, and compromised intestinal motility [4]. It is believed that EPI results from vagal denervation that causes loss of endogenous neuroendocrine signals that stimulate the pancreas to release digestive enzymes [5]. Therefore, EPI can result in micro and macro nutritional deficiencies.

In the UK and worldwide, there is an increasing pressure to provide high quality and timely interventions. However, due to limited specialist services available and a rising demand, it usually results in increased waiting times for patients to be seen and additional cost for increasing clinician appointments. AHP have shown to be effective in bridging service demand gaps [6], and can help reduce long waiting times for service for patients [7,8].

Currently, there are no standard guidelines in the UK for the biochemical monitoring and replacement of micronutrients in patients who have undergone esophagectomy. Bariatric surgery has been shown to have a profound effect on dietary and nutritional intake and the current British Obesity and Metabolic Surgery Society (BOMSS) guidelines recommend routine monitoring of biochemical, hematological and metabolic changes post bariatric surgery [9]. Due to the risk of nutritional deficiency posed by the nature of esophagectomy or subtotal/total gastrectomy, our unit recognized the need for long term monitoring of micronutrient levels in these patients. In our unit, we recently implemented an outpatient model using AHP, consisting of a cancer nurse specialist and specialist dietician to conduct routine follow-up for 5 year duration post esophagectomy or subtotal/total gastrectomy.

## 2 Materials and methods

### 2.1 Study population and setting

The AHP led outpatient clinic was established from February 2017. Patients who had undergone esophagectomy or subtotal/total gastrectomy for malignant tumors were seen at a consultant led clinic for their first review, which is two weeks after they had been discharged from hospital post-operation. All patients without ongoing post-operative complications or disease recurrence were eligible for AHP led clinic from their second visit. They were given an extended 30 min appointment in comparison to a traditional average of 20 min consultant appointment. Between February 2017 and February 2018, all patients who were eligible and who attended the AHP clinic at our esophago-gastric center, were included in our study, which is looking at a cross section of our patient population.

### 2.2 Baseline assessment and follow-up measurements

All patients who attended the clinic and met the eligibility criteria outlined above had micronutrient screening for up to 5 years post-surgery. In particular, the focus was on screening for ferritin, folate, vitamin B12 and vitamin D deficiencies. Additionally, patients were screened for EPI and malabsorption through series of questions related to symptoms of EPI including steatorrhea, flatulence, loose stools, urgency to defecate and difficulty flushing stools. All patients attending the AHP clinic were started on A-Z multivitamin tablets from their first visit. Furthermore, patients that reported symptoms indicative of EPI or malabsorption were started on 25,000 units or 40,000 units of Creon (pancreatic enzyme replacement therapy). Patients that were found to be deficient in Ferritin were given additional Ferrinject injection and started on ferrous sulphate. Patients found deficient in Vitamin B12, folate or vitamin D were given additional B12 injections, folic acid replacement and/or cholecalciferol, respectively.

All patients found deficient in any of the above mentioned micronutrients were invited for a follow-up measurement of the respective deficiencies, with the aim of retesting patients within a year's time. Patients not found deficient in any of the micronutrients were invited for an annual follow-up and were discharged from the AHP clinic 5 years post-surgery.

### 2.3 Definitions of micronutrient deficiencies

Patients were categorized as deficient in the following micronutrients according to our hospital trust guidelines and cut-off measurements:

• Serum ferritin levels <20 mmol/L
• Serum folate levels <3 mmol/L
• Serum vitamin D levels <25 mmol/L
• Serum vitamin B12 levels <180 mmol/L

### 2.4 Statistical analyses

Data were analyzed using SPSS 18 software (PASW 18.0 for Mac, SPSS Inc., Chicago, USA). Continuous variable values are presented as mean (±standard deviation). Average months for postoperative follow-up are presented as median values (range). Statistical significance level used was 0.05 and *P* values are two-tailed. Continuous variables were compared using student *t*-test (paired *t*-test) and missing data were taken in to account and deleted using a pairwise approach.

## 3 Results

### 3.1 Study cohort

Over a 12 month period (February 2017 to February 2018), 75 patients were reviewed in the AHP clinic having had surgery between 2013 and 2018. At a median follow-up of 18 (2–60) months, 63 of 75 patients (84%) were included in the study. As our center is a tertiary referral center, some patients were referred back to their regional center for future follow-ups after their initial review at our AHP clinic (n = 9) and hence not included in the study. Patients with disease recurrence (n = 2) and patients who died during this study period (n = 1) were not included. Clinical characteristics of the study cohort are shown in Table 1. Among the study cohort, 77.78% (n = 49) had undergone esophagectomy and 22.22% (n = 14) had undergone sub-total or total gastrectomy. Mean micronutrient levels at <12, 12-36 and 36-60 months post-operatively are displayed in Table 2.

**Table 1:** Characteristics of study cohort.

N	63
Mean age, years	68.05
Sex (Men/Women)	53(84.13%)/10(15.87%)
Operative approach	
Esophagectomy	49 (77.78%)
Sub-Total Gastrectomy	7 (11.11%)
Total Gastrectomy	7 (11.11%)
Median follow-up, months (range)	18 (2–60)

**Table 2:** Mean micronutrient levels Postoperatively.

	<12 months Postoperatively	12–36 months Postoperatively	36–60 months Postoperatively
Ferritin, mmol/L	92.87 ± 79.59	47.88 ± 49.44	37.13 ± 53.21
Folate, mmol/L	7.9 ± 5.10	9.61 ± 5.1	8.67 ± 4.77
Vitamin D, mmol/L	39.65 ± 22.38	46.29 ± 17.80	30.35 ± 24.68
Vitamin B12, mmol/L	357.32 ± 158.38	342.76 ± 178.99	355.57 ± 177.78

### 3.2 wt loss

The mean weight loss % at <12, 12–36 and 36–60 months post-operatively were 7.96% ± 5.50%, 7.62% ± 5.59% and 14.35% ± 6.46% respectively. The proportion of patients with clinical severe weight loss of >15% from their preoperative weight was 22.22% (n = 14). More than 15% weight loss was observed in 13.04% at <12 months, 11.76% at 12–36 months and 39.13% at 36–60 months follow-up. It is important to note that this is a cross-section of the patient population seen at the AHP clinic and as patients were seen at various months following their operation, it is not possible to draw a conclusion about a trend in weight loss.

### 3.3 EPI and malabsorption symptoms

The proportion of patients identified with symptoms of malabsorption or EPI at the initial AHP follow-up was 31.75% (n = 20). The proportion of patients with symptoms of malabsorption who underwent gastrectomy or esophagectomy were 25% (n = 5) and 75% (n = 15) respectively. All patients identified with symptoms of malabsorption were started on 25,000 units or 40,000 units of Creon depending on the severity of the symptoms. Fifteen of the 20 patients were started on 25,000 units of Creon and a further 5 patients were started on 40,000 units. Eighteen of the 20 patients that were started on Creon were followed-up. At a median follow-up of 7 months (3−13), 66.67% patients (n = 12) noticed settlement of symptoms of malabsorption with taking Creon supplements. Additionally, 16.67% (n = 3) noticed improvement in symptoms whilst a further 16.67% (n = 3) didn't notice any improvement and had ongoing symptoms of malabsorption and EPI.

### 3.4 Ferritin deficiency

The mean ferritin levels were 59.83 ± 68.27 mmol/L. Clinically severe ferritin deficiency was established to be < 20 mmol/L of serum ferritin. Proportion of patients reviewed with severe ferritin deficiency were 42.86% (n = 27). Mean ferritin levels for those 27 patients who were deficient was 12.85 ± 4.91 mmol/L. Proportion of patients with ferritin levels <20 mmol/L at <12 months follow-up was 26.09% compared to 52.17% at 36–60 months follow-up. 36 of the 63 patients were retested after the initial follow-up. 100% of patients that were deficient at the initial follow-up were retested. Patients were retested at a median of 7.5 months (2−14). Ferritin levels significantly improved after the initial follow-up as mean ferritin levels at re-test was 68.1 ± 85.74 mmol/L (*P* = 0.013). Proportion of patients that improved their ferritin levels at retest who were deficient initially was 85.19% (n = 23). Two patients were noted to be non-compliant with taking the multivitamins regularly and 1 patient died by the time of retest.

### 3.5 Folate deficiency

Mean folate level was 8.64 ± 5.03 mmol/L. Proportion of patients with severe folate deficiency of serum folate levels <3 mmol/L were 9.52% (n = 6). Twenty-one of the 63 patients (33.33%) were retested at a median of 10 months (3−14). Five out of 6 patients with deficiency were retested as 1 patient died. Mean folate levels at re-test is 11.30 ± 6.11 mmol/L showing a significant improvement from folate levels at the initial follow-up (*P* = 0.014).

### 3.6 Vitamin D deficiency

Mean serum vitamin D levels were tested for 60 patients. Vitamin D levels at initial AHP follow-up were not available for 3 patients. Mean serum vitamin D levels at initial AHP follow-up was 37.97 ± 23.09 mmol/L. Proportion of patients with vitamin D deficiency with serum vitamin D levels <25 mmol/L was 36.67% (n = 22). Twenty-eight of the 60 patients (46.67%) were retested at a median of 8 months (2−14). Proportion of patients with deficiency at initial AHP follow-up who were retested was 81.82% (n = 18). Three patients were not retested as they had missed their appointment and 1 patient died. Vitamin D levels improved significantly post initial AHP follow-up with mean of retested serum vitamin D level being 55.86 ± 24.49 mmol/L (*P* < 0.0001).

### 3.7 Vitamin B12 deficiency

Mean serum vitamin B12 levels at initial AHP follow-up was 352.66 ± 171.60 mmol/L. Proportion of patients with severe B12 deficiency was 6.35% (n = 4). Twenty-two of the 63 (34.92%) were re-tested with a median retest at 9.5 months (3−14). 100% of the patients with deficiency at the initial AHP follow-up were retested and showed improvement in their serum B12 levels. Mean serum B12 levels at retest was 356.27 ± 180.67 mmol/L and doesn't show a statistically significant improvement in B12 levels from the initial AHP follow-up (*P* = 0.079).

## 4 Discussion

There is growing evidence that with advancements in perioperative care and modern operating techniques, oncological outcomes have improved for patients in the post-operative period [10,11]. Due to these recent advancements in surgery, there has been a recent shift of focus towards the quality of life post esophago-gastric surgery [12]. Although there is a vast amount of data available on morbidity and mortality post esophagectomies, there are very few studies looking at the post-operative nutritional aspect which can in turn affect the overall survival and quality of life outcome. Increasing number of studies show that post esophagectomy or subtotal/total gastrectomy, patient's nutritional status can become severely compromised [2,3,5,12,13]. This can be worsened by the addition of more complex regimens of chemotherapy and radiotherapy; as for many patients a multimodal therapy is warranted to improve oncologic outcomes. A recent systematic review looking at nutritional consequences post-esophagectomy reported a weight loss of 5%–12% at 6 months postoperatively, with more than half of patients losing >10% of body weight at 12 months [2]. Although the prevalence of weight loss in patients post-esophagectomy was observed, the exact reason behind this is still poorly understood. Although weight loss could be a surrogate marker of nutritional deficiency, there have been very limited numbers of studies assessing the specific micronutrient deficiency post esophagectomy. A recent cohort study among 45 disease-free patients, found that at a median follow-up of 23 months, malnutrition and malabsorption are highly prevalent, with a reported incidence of EPI of 27% and a significant reduction in serum Vitamin A and E levels postoperatively [3].

In our study, a high proportion of patients had >15% weight loss at <12 months (13.04%), 12–36 months (11.76%) and at 36–60 months (39.13%) postoperatively. This figure is consistent with other studies including Heneghan et al. [3] who reported 39% of patients to have had more than 15% weight loss at 18–24 months postoperatively and Martin et al. [13] who reported 35% of patients to have had more than 15% weight loss at 3 years postoperatively. Additionally, due to vagal denervation during esophagectomy, EPI can occur due to loss of endogenous neuroendocrine signals to stimulate the secretion of pancreatic juices for absorption of nutrients [14]. The proportion of patients identified with symptoms of EPI were 31.75% at the initial follow-up. Amongst that 66.67% patients noticed settlement of symptoms of EPI with taking Creon supplements and an additional 16.67% noticed improvement in symptoms. As highlighted by Heneghan et al. [3], although symptoms of malabsorption and EPI are highly prevalent in patients post esophagectomy or gastrectomy, it is very much amenable. Our study shows that with patient compliance, symptoms related to EPI can be easily treated with Creon.

Our study also evaluates the specific nutritional deficiencies observed post esophago-gastric cancer surgery. A high proportion of our patients were found to be deficient in ferritin (42.86%) and Vitamin D (36.67%). Additionally severe folate (9.52%) and vitamin B12 (6.35%) deficiency were also observed in our study. This study highlights a statistically significant improvement in mean serum levels of Ferritin, Folate and Vitamin D at re-test following the initial follow-up and 100% of the patients with vitamin B12 deficiency at initial AHP follow-up showed improvement in their serum B12 levels at re-test. Several factors could contribute to malnutrition and malabsorption seen frequently after major upper gastrointestinal surgery. Permanent anatomical changes are present in patients who undergo esophagectomy. This can lead to an impaired gastrointestinal function with patients reporting symptoms such as early satiety, postprandial dumping and altered stool frequency postoperatively. This has an impact on the nutrient intake thereby leading to weight loss and nutritional deficiency as seen in our study. Additionally, anatomical alterations can have an impact and alter the bacterial growth in the intestines thereby affecting absorption of nutrients from the small intestine [15], with few studies showing prevalence of SIBO post total gastrectomy to be as high as 77% [16]. Although these are likely theories behind the reason for observing prevalence of nutritional deficiency in this group of patients, no clear cause has been identified.

In our study we identified that at least 60.3% of the patients had at least one micronutrient deficiency. Due to the high prevalence of severe micronutrient deficiencies, it is important to carefully screen for these deficiencies and treat them accordingly. Equally it is important to appreciate that in high volume tertiary NHS centers, there is an ever increasing demand for clinician outpatient appointment and compliance to such demand is becoming increasingly difficult with the addition of financial pressure within the NHS. Several studies have shown that nurse practitioners can provide safe and high quality services for patient to help improve the patient's overall course in hospital and in the outpatient settings [17,18]. Our study shows that AHP led clinics are safe and can provide high-quality and cost-effective services by carefully screening for nutritional deficiencies and appropriately treating them with adequate follow-up. Currently, an average of 44 AHP clinics are run in a year in our tertiary unit with an average of 7 patients per clinic. This frees up 308 consultations for clinicians or surgeons in a year which could be used for addressing and managing complex post-operative patients.

This study does have some limitations including a relatively small study sample size. There is also heterogeneity present amongst the patients in the study group including multimodal therapy received and the surgical approach. However, our study does provide statistically significant evidence behind the effectiveness of screening and appropriately treating the micronutrient deficiencies. Currently, there are no standard guidelines in the UK for biochemical screening of micronutrients in cancer patients post esophagectomy or gastrectomy. In our unit patients without ongoing post-operative complications or recurrence attend the AHP clinic and have biochemical micronutrient screening for up to 5 years post-surgery. Patients found deficient were started on replacement and are given an additional follow-up appointment to check compliance, resolvement of symptoms and re-testing the serum micronutrient levels.

In conclusion, this study highlights the prevalence of micronutrient deficiencies in cancer patients post esophagectomy and/or subtotal/total gastrectomy. AHP led clinics can provide safe, cost-effective and careful screening for micronutrients is necessary in these groups of patients to ensure that the deficiencies are corrected appropriately and thereby enable a better quality of life post esophagectomy or gastrectomy in cancer patients.

## Provenance and peer review

Not commissioned, externally peer reviewed.

## Ethics approval

Ethics requirement for this retrospective study was checked with NHS Health Research Authority (HRA) and ethics approval was not required for this study.

## Declaration of competing interest

The authors have none to declare.
